# The Effects of a Short-Term Combined Exercise Program on Liver Steatosis Indices and the Lipidemic and Glycemic Profile in NAFLD Individuals: A Pilot Study

**DOI:** 10.3390/metabo13101074

**Published:** 2023-10-13

**Authors:** Dimitrios Voudouris, Maria Horianopoulou, Zoi Apostolopoulou, Costas Chryssanthopoulos, Mari Bardopoulou, Maria Maridaki, Theodoros Vassilakopoulos, Michael Koutsilieris, Anastassios Philippou

**Affiliations:** 1 Department of Physiology, Medical School, National and Kapodistrian University of Athens, 11527 Athens, Greece; 2 Department of Physical Education and Sport Science, School of Physical Education and Sport Science, National and Kapodistrian University of Athens, 17237 Athens, Greece

**Keywords:** aerobic exercise, glucose tolerance, insulin resistance, intrahepatic lipid, resistance exercise, steatohepatitis

## Abstract

Non-alcoholic fatty liver disease (NAFLD) is a very common liver disease associated with obesity, unhealthy diet, and lack of physical exercise. Short-term aerobic or resistance exercise has been shown to result in reduced liver fat in patients with NAFLD; however, the impact of the combination of these types of exercise has received less attention. This study investigated the effect of a short-term (7 days) concurrent exercise training program performed daily on liver steatosis indices, as well as the glycemic and lipidemic profile of overweight/obese sedentary volunteers. Twenty adult patients (age: 47.3 ± 12.3 yrs, body mass index: 32.4 ± 3.4 kg/m^2^) with NAFLD, detected by ultrasound and hematological indices, participated in the study. Pre- and post-exercise intervention assessment included body weight (BW), waist circumference (WC), hip/waist ratio (H/W), Homeostasis Model Assessment Insulin Resistance (HOMA-IR), blood lipids, and steatosis indices. Fatty Liver Index, Lipid Accumulation Index, WC, H/W, triglycerides, and total cholesterol were improved (*p* < 0.05) post-exercise, while no differences (*p* > 0.05) were observed in BW, HOMA-IR, HDL, LDL, Hepatic Steatosis Index, and Framingham Steatosis Index compared to pre-exercise values. It is concluded that a 7-day combined exercise program can have beneficial effects on hepatic steatosis and central adiposity indices, independently of weight loss, in patients with NAFLD.

## 1. Introduction

Non-alcoholic fatty liver disease (NAFLD) is a condition in which fat accumulates in the hepatocytes (steatosis), with this fat accumulation not associated with chronic alcohol intake. Non-alcoholic steatohepatitis (NASH) is the next stage of NAFLD, where, in addition to steatosis, an inflammatory condition develops that gradually leads the hepatocytes to apoptotic cell death. As a chronic state of liver inflammation, NASH eventually results in liver fibrosis and, if the disease progresses, its pathological mechanisms drive the deterioration into the stages of liver cirrhosis and liver cancer [1,2]. The identification of the factors related to the stages of the disease, from NAFLD to fibrosis to liver cirrhosis, is summed up in obesity, insulin resistance, type 2 diabetes, some features of the metabolic syndrome, and age as it progresses [3].

It is noteworthy that there is no FDA-approved drug treatment for NAFLD and the therapeutic tools that have been proposed include regular physical activity, the adoption of a balanced diet, and weight loss courses [4]. Indeed, gradual changes in the patient’s lifestyle are key to reversing NAFLD pathology [5]. The combination of exercise and an appropriate diet, such as a low glycemic index Mediterranean diet, seems to produce the best result [6], although it has been reported that exercise interventions, such as high intensity interval training, may provide benefits in adolescents with NAFLD without following an appropriate dietary regime [7]. The beneficial effect of exercise may be intensity dependent [8]. Recently, the American College of Sports Medicine recommended 150 min moderate or 75 min vigorous physical activity per week for those who suffer from NAFLD, including the patients with cirrhosis [9].

The lifestyle changes usually lead to a reduction in body weight that seems to be a key factor to improve the condition. Specifically, research has shown that a 5% body weight loss with exercise and diet can reduce steatosis levels in NAFLD and reverse histological changes in stages of fibrosis and hepatocyte necrosis [10], while a reduction of only 3% of body weight in non-obese NAFLD patients leads to remission of the disease [11]. Interestingly, however, exercise alone appears to be an effective therapeutic intervention for NAFLD independently of weight loss, as its effects occur regardless of whether the patients eventually lose weight [12]. Indeed, the positive effects of physical activity/exercise on metabolic disorder- and NAFLD-related biomarkers are well documented by several studies [13], which used various exercise training protocols, utilizing aerobic or resistance exercise, and lasted from 2 to 52 weeks [14,15,16]; however, the impact of the combination of both types of exercise has received less attention [17]. In a recent meta-analysis, it was reported that, overall, exercise significantly reduced intrahepatic fat, low density lipoprotein cholesterol, triglycerides, aspartate aminotransferase (AST), and alanine aminotransferase (ALT), without engendering a significant weight loss [17]. Moreover, only a few short-term exercise interventions, lasting less than two weeks, have been conducted in patients with NAFLD. Specifically, those studies included a 7-day aerobic exercise training and revealed remarkable effects on biomarkers related to NAFLD [18,19,20,21]. Interestingly, however, a small group of six obese participants unaffected by NAFLD showed no change in biomarkers related to NAFLD after a 7-day exercise program [21]. Bearing in mind the fact that NAFLD patients exhibit low levels of physical activity, while adherence to exercise interventions is a key issue [9], a beneficial effect of such a short-time exercise program might be motivational, leading to increased physical activity. Thus, this study aimed to investigate the potential effects of a combined (aerobic and resistance), short-term exercise program, performed daily, on liver steatosis indices, and the glycemic and lipidemic profile of overweight/obese sedentary patients with NAFLD.

## 2. Materials and Methods

### 2.1. Participants

Twenty adult volunteers, twelve men and eight women, were recruited from a private medical clinic; participants were all overweight or obese (body mass index-BMI > 25 kg·m^−2^) and diagnosed with NAFLD by ultrasound and blood biomarkers, while 10 of them were under pharmacological treatment for coexisting metabolic dysfunctions (diabetes, dyslipidemia, etc.). In particular, three participants were receiving a single therapy, four participants were receiving double, two participants triple, and one participant quadruple pharmacological therapy. Throughout the study, these patients continued to take their medication as prescribed by their physician (Table 1). Furthermore, five volunteers were hypertensive and two of them also suffered from type 2 diabetes. One of the two diabetics used insulin for treatment and, therefore, was excluded from the oral glucose tolerance test (OGTT), as well as from fasting glucose and insulin measurements.

The volunteers signed an informed consent form for their participation in the study, after they had been thoroughly informed about the purpose of the study, the experimental procedures, or any possible discomfort and risks involved. All patients were examined by a physician (medical history, physical examination, resting ECG, and carotid and heart ultrasound) and a medical clearance was provided before exercise testing. The study had received the approval of the Ethical Committee of the University and all procedures were in accordance with the Helsinki declaration of 1975, as revised in 1996.

### 2.2. Experimental Design—Procedures

After a 12 h overnight fast, participants underwent baseline assessment that included hepatic ultrasound, biochemical blood testing, and OGTT. On the following day, somatometric measurements were taken and the volunteers performed an incremental cycling exercise test to volitional fatigue to establish a workload–heart rate (HR) relationship to be used for the exercise training. Afterwards, participants performed three different resistance exercises to estimate their individual one-repetition maximum (1-RM). For the next 7 consecutive days, volunteers followed a concurrent exercise training program (see details below). On the day following completion of the training program, again after a 12 h overnight fast, hepatic ultrasound, blood testing, OGTT, and somatometric measurements were repeated.

#### 2.2.1. Baseline Measurements

After a 12 h overnight fast, participants arrived at the laboratory between 7:30–08:00 a.m. and hepatic ultrasound (GE Logiq 7, General Electric Co., California, CA, USA) examination was performed by an experienced physician who was not aware (blind) of the purpose of the study. Then, baseline blood sampling and the OGTT were performed. Specifically, with the participants sitting quietly, a 10 mL venous and duplicated 25 μL of capillary blood samples were collected. Afterwards, 75 g of glucose (D-Glucose Anhydrous, Sigma-Aldrich, Miami, MI, USA) dissolved into 300 mL of water were administered to the participants. At 15, 30, 45, 60, 90, and 120 min after glucose ingestion, duplicate 25 μL of capillary blood samples were collected from each volunteer. On the following day, participants visited the lab after having their last meal at least 4 h earlier and body mass (Tanita BC 545n, Manchester, UK), height (Seca 216, Hamburg, Germany), and waist and hip circumference (using a flexible tape) were measured. Then, volunteers performed an incremental cycling exercise test (Technogym, Artis, Cesena, Italy) to fatigue to determine the exercise intensity equivalent to 75–80% of HR maximum (HRmax) to be used for the aerobic exercise training. Heart rate was measured by telemetry (Polar T31, Kempele, Finland). The initial workload was 30 watts for 3 min and exercise intensity was increased by 30 watts every 3 min until participants were unable to maintain a pedal speed of 50 revolutions/min. Using regression analysis, the exercise intensity corresponding to 75–80% of HRmax was estimated. After a 15 min passive recovery, participants practiced the correct execution technique before performing three basic exercises (Lat Pull Down, Landmine Press, and Goblet Squat) in resistance exercise machines (Amila BodyCraft—PFT 44709, Athens, Greece) to estimate 1-RM. A 10 min rest was allowed between each 1-RM estimation cycle. In the 1-RM estimation testing, three sets were used, one for warm-up (8–10 repetitions) with a light weight, one at 50–70% of perceived 1-RM, and one set for 1-RM estimation, with 2 min rest between the first and second set and 3 min between the second and third set. In the 1-RM estimation set, volunteers were instructed to exert maximal effort, and no more than 8 repetitions (range 4–8 repetitions) were performed by any participant. The weight lifted and the repetitions achieved were used to estimate 1-RM [22]. After completing the 1-RM estimation testing for the first resistance exercise, volunteers were familiarized with the correct execution of the other two exercises included in the resistance exercise program of the study. Post-intervention measurements, i.e., blood sampling, OGTT, and somatometric measurements, were performed on the day following completion of the 7-day exercise training, following procedures identical to the baseline measurements.

#### 2.2.2. Exercise Training Program

All participants followed a supervised exercise training protocol that combined aerobic and resistance exercise for 7 consecutive days. Each exercise session lasted for 60 min, beginning with a 5 min warm-up cycling at 60% HRmax, followed by 20 min of resistance exercise at 65–75% of 1-RM and 30 min of aerobic exercise at 75–80% of HRmax performed on a cycle ergometer (Technogym, Artis, Cesena, Italy), and completed with a 5 min cool down. Regarding resistance training, each day, volunteers performed in circuit four exercises targeting one of the following main muscle groups: chest (landmine press, cable cross over, dumbbell pull over, dumbbell lateral raise), back (lateral pull down, dumbbell rowing, cable reverse fly, superman back extension), or lower limbs (dumbbell goblet squat, dumbbell glut bridge, hip abduction with resistance band, hip adduction with fitness ball), completing two sets of 10 repetitions each, with 1 min rest between sets. A broad range of equipment was used, i.e., cables, fitness ball, resistance bands, dumbbells, and exercise mat. On day 4 of the exercise intervention, patients followed a milder exercise protocol consisting only of 30 min aerobic exercise at 75–80% of HRmax, while on days 5, 6, and 7 the exercise protocols of day 1, 2, and 3 were repeated, respectively.

#### 2.2.3. Blood Samples Analysis

Capillary blood samples were dispensed into Eppendorf vials containing 250 μL of 2% perchloric acid, mixed well, centrifuged at 1500× *g* for 4 min and stored at −80 °C for further analysis. The two deproteinized supernatants obtained for each time point were analyzed in duplicate on a microplate reader (Molecular Devices, Versa max, San Jose, CA, USA) for blood glucose (Randox Laboratories Ltd., Crumlin, UK). Using the trapezoidal rule, the total area under the blood glucose–time curve (AUC) was calculated from the blood glucose data obtained at the different time points during the OGTT. A total of 2 mL from the venous blood sample was used for the determination of platelets (PLT) count and glycated hemoglobin (HbA1c) (Spinreact S.A.U., Girona, Spain). The remaining venous sample was left to clot for 30 min and centrifuged at 1500× *g* for 10 min. Serum was separated and divided into aliquots of 500 μL and stored at −80 °C for subsequent analysis, with the exception of one aliquot that was used fresh for the determination of low-density lipoprotein (LDL) cholesterol (Spinreact S.A.U., Girona, Spain), according to the manufacturer’s directions, on an automatic analyzer (Spin 200E, Spinreact S.A.U., Girona, Spain). Serum insulin was determined using an electrochemiluminescence immunoassay kit (Elecsys Insulin, Roche Diagnostics, Indianapolis, IN, USA) on an automatic analyzer (Cobas e411, ROCHE Diagnostics GMBH, Mannheim, Germany). Serum AST, ALT, γ-glutamyl transferase (γ-GT), C-reactive protein (CRP), albumin (ALB), glucose, triglycerides, total cholesterol (TC), and high-density lipoprotein (HDL) cholesterol were analyzed on an automatic analyzer (Spin 200E, Spinreact S.A.U., Girona, Spain), using commercially available kits (Spinreact S.A.U., Girona, Spain).

#### 2.2.4. Insulin Resistance and Liver Steatosis and Fibrosis Indices

Before and after the exercise training intervention, the homeostatic model assessment for insulin resistance (HOMA) [23], the hepatic steatosis index (HSI) [24], the fatty liver index (FLI) [25], the Framingham steatosis index (FSI) [26], the lipid accumulation product (LAP) [27], the FIB-4 score (FIB-4) [28], the NAFLD fibrosis score (NFS) [29], and the AST/ALT ratio (AAR) [30] were determined.

#### 2.2.5. Diet and Physical Activity Control

Participants were required to refrain from any physical activity two days before the baseline measurements and follow the same diet on the day before baseline blood testing and post-intervention blood testing. In addition, volunteers were not allowed to participate in any physical activity during the intervention period other than the supervised exercise training program of the study.

### 2.3. Statistics

Data were analyzed using SPSS (SPSS Inc., Chicago, IL, USA version 23). The Shapiro–Wilk test was used to assess the normality of the data. The Wilcoxon Signed Rank test was used to examine the effect of exercise intervention (pre- vs. post-intervention measurements) on HbA1c, AST, γ-GT, CRP, FIB-4, HDL, AST/ALT ratio, waist and hip circumference, HOMA index, and the hepatic steatosis FLI and fibrosis NFS indices. The remaining variables were analyzed using a two-tailed paired t-test. Effect size was estimated by calculating Cohen’s d (d) in t-tests and r in Wilcoxon Signed Rank tests. In case of significance, 95% confidence intervals (CI) of the means (t-test) and medians (Wilcoxon) differences were also reported. To estimate the sample size, G*Power v. 3.1.9.7 was used. Utilizing the HOMA-IR model data from the study by Haus and colleagues [19], before and after a short 7-day exercise program in NAFLD patients, for a medium effect size of 0.7, a power of 0.8, and a probability level of 0.05, an estimated sample size of 19 was revealed. Data are presented as mean ± SD, and the level of significance was set at *p* < 0.05.

## 3. Results

The effects of a short-term, daily, concurrent exercise program on liver steatosis indices, as well as on glycemic and lipidemic profile of overweight/obese patients with NAFLD, were examined in this study.

There were no significant changes in HDL (Table 2) and fasting plasma glucose levels (Pre: 96 ± 18 mg/dL vs. Post: 99 ± 20 mg/dL; *p* = 0.456; *n* = 19) at the end of the 7-day exercise intervention compared to baseline measurements in the NAFLD patients. Similarly, no significant improvements were found in HbA1c levels (Pre: 5.5 ± 0.6% vs. Post: 5.4 ± 0.7%; *p* = 0.19; *n* = 19), fasting insulin levels (Pre: 8.6 ± 5.0 mIU/L vs. Post: 8.1 ± 4.6 mIU/L; *p* = 0.339), and the HOMA index (Pre: 2.16 ± 1.63 vs. Post: 2.13 ± 1.61, *p* = 0.49; *n* = 19) after the completion of the 7-day intervention. Similarly, no changes were observed in AST, ALT, γ-GT, CRP, and albumin levels as a result of the 7-day exercise intervention program (Table 2). Furthermore, mean blood glucose in the OGTT was not different (Pre: 129 ± 31 mg/dL vs. Post: 123 ± 27 mg/dL; *p* = 0.14; *n* = 19), and neither was the AUC (Pre: 15,470 ± 4059 mg/dL/2h vs. Post: 14,570 ± 3515 mg/dL/2h; *p* = 0.11; *n* = 19).

Interestingly, however, significant improvements were observed in triglycerides, which also probably contributed to the observed reduction in total cholesterol post-exercise. Furthermore, reductions were found in waist circumference, hip circumference, and in the waist/hip ratio after the exercise intervention, though no significant improvement was found in the patients’ body weight (Table 2).

Moreover, hepatic steatosis indices LAP and FLI significantly improved after the 7-day exercise program, while no significant changes were found in the hepatic steatosis indices HIS and FSI, as well as in hepatic fibrosis indices FIB-4, NFS, and AAR at the end of the short-term exercise intervention (Table 3).

## 4. Discussion

This study investigated the potential changes in liver steatosis indices, as well as in the glycemic and lipidemic profile of overweight/obese patients suffering from NAFLD, following a short-term concurrent training program performed daily, without any accompanying dietary intervention or intentional body weight loss. The main finding of the study was that a 7-day exercise program that combines aerobic and resistance components can improve hepatic steatosis and central adiposity indices in patients affected by NAFLD regardless of body weight loss. Since advanced fibrosis was not present in the participants of this study based on the baseline scores of hepatic fibrosis indices FIB-4, NFS, and AAR, no significant changes were found in these scores after the completion of the short-term exercise training, as expected.

Specifically, after the one-week exercise intervention, hepatic steatosis, as assessed by the indices LAP and FLI, improved, while other indices such as HSI and FSI showed no significant improvement. The fact that each steatosis index is based on different variables may explain this “partial” improvement of steatosis in this study. LAP and FLI indices are based on triglycerides levels and waist circumference, which were found to improve following exercise training, while HSI and FSI evaluate glycemic and metabolic factors as well, such as the presence of type 2 diabetes. In addition, the reduction in triglycerides, in turn, had an influence on total cholesterol, which also decreased after the exercise intervention. Nevertheless, the significant decline in indices associated with hepatic steatosis found in this study is of major importance, since it is also associated with a reduction in risk of cardiovascular complications [27,31] which are the leading cause of death in NAFLD patients without hepatic fibrosis [32]. The reduction in serum lipids such as triglycerides and total cholesterol post-exercise training found in this study could also be the result of an increased uptake and use by the liver resulting in their reduced availability in the circulation [33]. Furthermore, it is well known that regular exercise reduces inflammation and oxidative stress which promote hepatic cell death and tissue injury, thus contributing to the pathophysiology of NAFLD and NASH [34,35]. Unfortunately, no such factors were measured in the present study; however, Fealy and colleagues demonstrated a reduction in markers of hepatocellular apoptosis after a 7-day aerobic program in NAFLD patients [18].

Although the combination of resistance and aerobic exercise would be expected to result in an improvement of the glycemic profile of the patients, no significant alterations were found in this study. Specifically, the levels of fasting plasma glucose and fasting insulin, as well as HOMA-IR index, did not change after the completion of the 7-day exercise program. Regarding the effect of a short 7-day exercise program on fasting plasma glucose and insulin concentrations in NAFLD patients, results are conflicting even for similar studies from the same laboratory, reporting either no change [18,19] or an improvement [20,21]. On the other hand, after a daily/weekly aerobic program, HOMA-IR has been found to have decreased [19,20], indicating reduced insulin resistance; blood glucose AUC in the OGTT was also lower, and insulin sensitivity measured by various methods also showed improvement [18,19,20,21]. However, it is interesting to note that an aerobic program of 7 consecutive days had no effect on any of the metabolic factors studied in six obese (two men and four women) that did not have NAFLD [21]. In the present study, AUC in the OGTT showed a tendency to improve (*p* = 0.11) following the exercise intervention. We assume that the combination of aerobic and resistance training utilized in this study might cause a higher metabolic stress, as opposed to the aerobic exercise training used in the previous short-term protocols that failed to engender clear glycemic benefits after the short exercise intervention. These glycemic responses might also be a result of possible muscle damage which the resistance exercise could have caused the sedentary patients of our study. Indeed, previous studies have shown a reduction in insulin sensitivity because of a strong inflammatory response and a decrease in glucose transporter GLUT4 following exercise-induced muscle damage [36], which may result in a “protective”, temporary state of insulin resistance to the high mechanical and metabolic stress and the consequent muscle damage [37,38,39,40].

Another reason for the discrepancy between the present and the other studies that have also employed a short 7-day exercise protocol in NAFLD patients [18,19,20,21] may also be the component of aerobic exercise. In the present study, volunteers exercise for only 30 min each day at 75–80% HRmax, while the other studies employed a longer aerobic component of 60 min at a higher exercise intensity of 85% HRmax [18,19,20,21]. It should be clarified, however, that, had the study been of longer duration, beneficial results in glycemic and lipidemic profile of the NAFLD patients would have occurred, as previous studies have demonstrated [9,17]. More studies are needed to reveal the potential role of the intensity of aerobic and resistance exercise in short-term, concurrent exercise training programs for the regulation of glycemic and lipemic profile of overweight/obese sedentary patients with NAFLD.

In terms of somatometric characteristics, body weight and BMI did not change in the overweight/obese patients with NAFLD after the completion of the short-term exercise protocol. This finding has also been observed in other studies of similar duration [18,19,20,21]. However, the waist and hip circumference, as well as the waist/hip ratio, was found to have decreased. As the body weight of the participants remained constant, we speculate that these changes in circumferences post-training could be attributed to the cell swelling effect, a physiological response that involves osmotic movement of water from the extracellular space to the inside of muscle cells to trigger protein synthesis and prevent proteolysis in muscle tissue [41,42,43]. Future research utilizing body composition assessment, e.g., by dual x-ray absorptiometry (DEXA), could contribute to the identification of the physiological mechanisms and the characterization of these somatometric changes in response to short-term exercise training.

The main limitation of the present study was that there was no control group. Ideally, a group of obese individuals with similar BMI and NAFLD indices to the exercise group should have been evaluated without participating in any form of exercise or physical activity. Some of the metabolic indices examined were in fact interconnected, influencing each other, such as triglycerides and total cholesterol. In addition, the examination of inflammatory cytokines and oxidative stress indices would have provided valuable information regarding the effects of a short-exercise combination program on these factors. Furthermore, a larger sample size would have added more power to the data obtained. Finally, the use of magnetic resonance spectroscopy would have provided important information regarding intrahepatic and visceral fat content.

## 5. Conclusions

In this study, we provided information about changes in liver steatosis, glycemic, lipidemic, and somatometric indices in response to a daily, concurrent exercise training program in patients with NAFLD. These changes could be potentially involved in a complex network of physiological processes associated with exercise-induced metabolic adaptations in overweight/obese patients. We revealed that a 7-day combined exercise training may be beneficial with respect to hepatic steatosis and central adiposity indices, independently of weight loss of the patients. More studies are needed to further characterize the physiological signature of metabolic adaptations to short-term exercise training in humans. Such a characterization could contribute to a better understanding of the adaptation mechanisms following exercise in patients with metabolic disorders and to the development of treatment strategies targeting the various components of the aforementioned network of adaptive physiological processes. Indeed, the ability of metabolic mechanisms to rapidly adapt to daily exercise might have clinical applications, as a short-term concurrent exercise that would directly trigger beneficial effects on hepatic steatosis and lipid metabolism could be used as an effective strategy to manage lipid disorders.

## Figures and Tables

**Table 1:** Type of drug/active substances per patient receiving medication during the study.

No of Patient	Type of Drug/Agent	Active Substance (AS)
2	SSRI, anti-inflammatory	escilatopram, mesazaline
4	anti-hypertensive, anti-inflammatory, anti-hyperlipidemic	ramipril, acetylsalicylic acid atorvastatin
5	anti-hypertensive	nebivolol
6	anti-hyperlipidemic, anti-hypertensive	simvastatin, olmesartan medoxomil
7	anti-hypertensive, anti-inflammatory, anti-hyperlipidemic	furosemide, acetylsalicylic acid, bisoprolol, ramipril, amlodipine besylate, rosuvasta
8	anti-hypertensive	olmesartan medoxomil, amlodipine, hydrochlorothiazide
9	anti-inflammatory, hypothyroidism	acetylsalicylic acid, Levothyroxine sodium
12	anti-diabetic, anti-hypertensive, anti-inflammatory, anti-hyperlipidemic	insulin degludec-iraglutide, insulin glulisine, acetylsalicylic acid, betaxolol, atorvastatin, olmesartan medoxomil
16	anti-hyperlipidemic, anti-hypertensive	amlodipine-valsartan, fenofibrate
19	antidepressants	citalopram

**Table 2:** Somatometric characteristics and serum biochemical markers before and after the 7-day exercise intervention in patients with NAFLD (mean ± SD; *n* = 20).

Variable	Pre-Intervention	Post-Intervention	Statistics
Age (yrs)	47.3 ± 12.3		
Waist circumference (cm)	113.2 ± 10.8	110.4 ± 10.8	P = 0.000 d = 1.67 CI: 1.96–3.49
Hip circumference (cm)	113.3 ± 8.5	112.5 ± 8.6	P = 0.007 Z value = −2.68 r = 0.42 CI: 0.21–1.24
Waist/Hip ratio	1.00 ± 0.09	0.98 ± 0.1	P = 0.000 d = 0.92 CI: 0.01–0.03
Body weight (kg)	97.1 ± 18.5	96.9 ± 18.6	P = 0.392 d = 0.20
BMI (kg/m^2)^	32.4 ± 3.4	32.39 ± 3.4	P = 0.397 d = 0.19
Triglycerides (mg/dL)	135 ± 50	116 ± 45	P = 0.046 d = 0.51 CI: 1.18–35.12
Total cholesterol (mg/dL)	195.8 ± 41.8	186.9 ± 31.8	P = 0.039 d = 0.50 CI: 0.50–17.30
HDL (mg/dL)	45.0 ± 13.1	43.3 ± 12.7	P = 0.17 Z value = −1.369 r = 0.22 CI: −0.67–3.93
LDL (mg/dL)	124.0 ± 37.7	120.9 ± 30.5	P = 0.392 d = 0.20 CI: −4.24–10.34
AST (U/L)	24.5 ± 11.1	26.0 ± 8.2	P = 0.38 Z value = −0.886 r = 0.27 CI: −7.10–4.27
ALT (U/L)	31.4 ± 16.8	29.7 ± 9.2	P = 0.561 d = 0.13 CI: −4.56–8.15
γ-GT (U/L)	42.5 ± 69.5	29.2 ± 30.9	P = 0.10 Z value = −1.631 r = 0.26 CI:-5.08–31.68
CRP (mg/dL)	2.6 ± 4.5	1.6 ± 1.9	P = 0.24 Z value = −1.165 r = 0.18 CI: 0–2
Albumin (g/dL)	4.4 ± 0.3	1.6 ± 1.9	P = 0.271 d = 0.25 CI: −0.27–0.08

ALT: alanine transaminase; AST: aspartate transferase; BMI: body mass index; CI: confidence interval; CRP: C-reactive protein; γ-GT: gamma-glutamyl transferase; d = Cohen’s d; HDL: high-density lipoprotein cholesterol; LDL: low-density lipoprotein cholesterol; P = probability level; r = effect size for the Wilcoxon Signed Rank test.

**Table 3:** Steatosis and fibrosis indices before and after the 7-day exercise intervention in the patients with NAFLD (mean ± SD; N = 20).

Variable	Cut-off Point	Pre-Intervention	Post-Intervention	Statistics
HSI	<30 >36	43.7 ± 4.90	42.63 ± 4.0	P = 0.234 d = 0.28 CI: −0.76–2.94
FLI	≤30 ≥60	77.3 ± 18.41	71.80 ± 22.3	P = 0.004 Z value = −2.91 r = 0.46 CI: 1.38–9.52
FSI	<23 ≥23	93.4 ± 60.90	103.85 ± 79.41	P = 0.510 d = 0.15 CI:
LAP	<20 ≥80	76.9 ± 30.20	63.17 ± 27.2	P = 0.009 d = 0.65 CI: 3.91–23.51
FIB-4	≤1.3 ≥2.67	0.97 ± 0.49	1.03 ± 0.6	P = 0.09 Z value = −1.68 r = 0.27 CI: −0.33–0.21
NFS	<−1.455 >0.676	−0.80 ± 0.96	−0.88 ± 0.9	P = 0.71 Z value = −0.37 r = 0.06 CI: −0.28–0.45
AAR	>0.80	0.87 ± 0.30	0.90 ± 0.2	P = 0.20 Z value = −1.27 r = 0.20 CI: −0.19–0.13

AAR: AST/ALT ratio; CI: confidence interval; d = Cohen’s d; FIB-4: FIB-4 score; FLI: fatty liver index; FSI: Framingham steatosis index; HSI: hepatic steatosis index; LAP: lipid accumulation product; NFS: NAFLD fibrosis score; P = probability level; r = effect size for the Wilcoxon Signed Rank test.

## Data Availability

All data included in this study are available upon request by contact with the corresponding author. Data is not publicly available due to privacy or ethical restrictions.
